# Genetic algorithm-based feature selection with manifold learning for cancer classification using microarray data

**DOI:** 10.1186/s12859-023-05267-3

**Published:** 2023-04-08

**Authors:** Zixuan Wang, Yi Zhou, Tatsuya Takagi, Jiangning Song, Yu-Shi Tian, Tetsuo Shibuya

**Affiliations:** 1 Division of Medical Data Informatics, Human Genome Center, Institute of Medical Science, The University of Tokyo, Tokyo, 108-8639 Japan; 2 Beijing International Center for Mathematical Research, Peking University, Beijing, 100871 China; 3 Graduate School of Pharmaceutical Sciences, Osaka University, 1-6 Yamadaoka, Suita, Osaka 565-0871 Japan; 4 Biomedicine Discovery Institute and Monash Data Futures Institute, Monash University, Melbourne, VIC 3800 Australia

**Keywords:** Cancer classification, Microarray data, Gene selection, Genetic algorithm, Manifold algorithm

## Abstract

**Background:**

Microarray data have been widely utilized for cancer classification. The main characteristic of microarray data is “large p and small n” in that data contain a small number of subjects but a large number of genes. It may affect the validity of the classification. Thus, there is a pressing demand of techniques able to select genes relevant to cancer classification.

**Results:**

This study proposed a novel feature (gene) selection method, Iso-GA, for cancer classification. Iso-GA hybrids the manifold learning algorithm, Isomap, in the genetic algorithm (GA) to account for the latent nonlinear structure of the gene expression in the microarray data. The Davies–Bouldin index is adopted to evaluate the candidate solutions in Isomap and to avoid the classifier dependency problem. Additionally, a probability-based framework is introduced to reduce the possibility of genes being randomly selected by GA. The performance of Iso-GA was evaluated on eight benchmark microarray datasets of cancers. Iso-GA outperformed other benchmarking gene selection methods, leading to good classification accuracy with fewer critical genes selected.

**Conclusions:**

The proposed Iso-GA method can effectively select fewer but critical genes from microarray data to achieve competitive classification performance.

**Supplementary Information:**

The online version contains supplementary material available at 10.1186/s12859-023-05267-3.

## Introduction

DNA microarray data have important applications in clinical decision support, such as diagnosis of disease (e.g., cancer) and prediction of clinical outcomes [1–3]. In recent decades, advances in DNA microarrays have enabled researchers to have a global view of cells. DNA microarray can measure the expression of thousands of genes simultaneously and help researchers to investigate the biological state of a cell [4]. Such high-throughput expression profiling can be used to distinguish a subject sample with cancer from those without or to classify tumor samples into different grades of cancer [1, 3]; these two applications are called *cancer classification* in this article. Due to the high expense of collecting microarray data with high-dimensional feature space ($p$), only limited data samples ($n$) are available from the population of subjects, which leads to the issue of *curse of dimensionality*, also known as the “*large p, small n*” problem [5, 6]. The high-dimensional gene feature space causes conventional statistical methods invalid. Even if some methods can handle the high-dimensional data, the inclusion of genes not related to cancer can deteriorate the accuracy of cancer classification [6, 7]. Thus, selecting a subset of genes relative to the cancer classification from microarray data (i.e., dimensionality reduction) is crucial and a pressing need. Various methods of performing dimensionality reduction have been proposed, and these methods can be generally grouped into feature extraction and feature selection [5]. Feature extraction methods project or compress the original features to create fewer new variables. The major drawback of these methods is that the interpretability of the variables can be lost during the projecting process. Alternatively, feature selection methods identify the most critical subset of features by removing the noisy features from the entire microarray data; thus, the characteristic and interpretability of data are preserved. Hira and Gillies [5] provided more detailed discussions on the advantages and disadvantages of these methods.

Feature selection can be considered as an optimization problem and include four groups: filter, wrapper, hybrid and embedded methods [8]. In recent decades, wrapper feature selection methods with meta-heuristics as search strategies have become increasingly popular in microarray data analysis [8, 9]. Meta-heuristic algorithms have advantages in fast convergence, excellent search ability, and high population diversity. They are superior to other methods in readability and interpretability and avoid premature convergence or falling into local optima [10]. On microarray data, meta-heuristics-based methods can search the optimal subset of genes more efficiently by using a specific fitness function to evaluate the candidate subsets of genes, and these methods can be combined with many classifiers for cancer classification [10]. Recently, many enhancements of the meta-heuristic algorithms have been proposed by mimicking the behaviors of organisms in nature. For example, artificial bee colony (ABC) [11, 12], cuckoo search (CS) [13, 14], bacterial colony optimization (BCO) [15], chimp optimization algorithm (ChOA) [16], forest optimization algorithm (FOA) [17], and genetic algorithm (GA) [18]. These enhancements are based on bio-inspired optimization [19] and showed good performance in gene selection [10]. However, individual algorithm usually has inherent limitations. Thus, the hybrid feature selection method is usually adopted to achieve better performance [20, 21]. A hybrid method combines filter- and wrapper-based methods for feature selection. Therefore, the hybrid method typically achieves the high accuracy characteristic of wrappers and the high efficiency characteristic of filters [22]. Meta-heuristic algorithms can be hybridized with feature extraction methods (e.g., the hybridization between ABC and independent component analysis [23]) or optimization methods (e.g., the binary particle swarm optimization and sine cosine algorithm [24]). Many meta-heuristics-based hybrid methods adopted GA, a method inspired by the evolutionary process of natural selection, to improve performance in feature selection [20, 25, 26]. For example, Alshamlan et al. [27] developed the genetic bee colony (GBC) algorithm by combining GA with the ABC algorithm, [28]. Das et al. embedded the Harmony Search (HS) algorithm with GA [29]. However, the present meta-heuristic-based hybrid methods have several shortcomings:

1. *Classifier dependency*: These methods use fitness values that include the classification accuracy of a specific classifier, which can lead to classifier dependency because the meta-heuristic algorithm aims to optimize the classification accuracy [30, 31].
2. *Randomness*: In the pre-experiments, it was found that even when the same algorithms and objective functions are used on the same dataset, randomness in the algorithms could result in quite different subsets of genes being selected when the analysis is repeated. Thus, it is necessary to employ a feature selection method that reduces the impact of algorithmic randomness.
3. *Linear space assumption*: Most meta-heuristics methods use linear distances to evaluate candidate subsets of genes. For example, Garro et al. [32] introduced a classification method that utilizes the ABC algorithm with a classification error function for feature selection and multiple artificial neural networks to evaluate gene subsets. This approach is based on the assumption that gene expression vectors are distributed in linear Euclidean space. However, this assumption does not always hold in practice [20]. Since genes are dynamically linked with each other, it is reasonable to assume that gene expression features lie in the nonlinear space. Thus, nonlinear algorithms, such as manifold learning, should be more appropriate for dimensionality reduction and fitness evaluation [33]. Among the nonlinear manifold learning methods, Isometric feature mapping (Isomap) has good performance in preserving the underlying data structure and could improve the classification accuracy [34, 35].

To solve the aforementioned issues, we propose a method called Iso-GA, which hybrids Isomap and GA to select the *optimal subset of genes*, i.e., the genes most helpful to cancer classification. The key ideas in the proposed method are as follows. Isomap is used to map high-dimensional nonlinear microarray data to a low-dimensional linear space. The correlation of gene subsets and cancer subtypes is measured by the Davies–Bouldin (DB) index [36] to reflect the clarity of division between samples of different classes in the mapped dataset. A feature selection framework with Iso-GA inserted is proposed to reduce the influence of randomness. In this framework, the GA search is repeated several times to select feature subset that optimizes the fitness function, and a new set containing the common features selected over a specified threshold number of times is used in the final classifier. The threshold is calculated based on the binomial distribution and the entire number of genes in microarray data. The threshold ensures that Iso-GA could select reasonable numbers of cancer-related genes from various *p*-dimensional datasets. By selecting a smaller subset of genes, the proposed method expects to improve cancer classification accuracy on microarray data.

## Methods

### Notation

The dataset adopted in this study can be denoted as $\left(X, {\varvec{y}}\right)=\{({{\varvec{x}}}_{{\varvec{i}}}, {y}_{i})|i=1,\dots ,n\}$, where ${{\varvec{x}}}_{{\varvec{i}}}=({x}_{i1},\dots ,{x}_{ip})$, ${\varvec{y}}={\left({y}_{1},\dots ,{y}_{n}\right)}^{T}$, and ${y}_{i}\in \left\{1,\dots ,C\right\}$ indicates the class label of ${{\varvec{x}}}_{i}$ where $C$ denotes the number of classes. Let *p* be the total number of features and *n* be the total number of samples.

In five fold cross-validation, we chose one of five folds in turn as a test set $({X}_{te}, {{\varvec{y}}}_{te})$ each time, and the other four folds as the training set $({X}_{tr}, {{\varvec{y}}}_{tr})$. For each training set, we generated the ${b}^{\mathrm{th}}$ bootstrap samples $({X}_{tr}^{\left(b\right)}, {{\varvec{y}}}_{tr}^{\left(b\right)})$ and $({X}_{val}^{\left(b\right)}, {{\varvec{y}}}_{val}^{\left(b\right)})$ as the training and validation sets, respectively.

Let each candidate solution be ${{\varvec{s}}}_{i}$, $i=1, \dots , pop.size$ (population size), and $|{\varvec{s}}|$ be the size of the solution.

### Isometric feature mapping (Isomap)

In 2000, Tenenbaum et al. [37] proposed a framework that uses the local metric information to learn the underlying global geometry of the data for nonlinear dimensionality reduction, referred to as Isomap. Isomap is a generalization of the conventional multidimensional scaling (MDS) algorithm for nonlinear manifolds [35]. MDS preserves the Euclidean distance between the data points consistent in the observation space and the target space as much as possible and assumes that the manifold is linearly or approximately linearly embedded in a high-dimensional observation space [38]. It attempts to maintain the geodesic distance on the manifold of the high-dimensional observation space consistent with the Euclidean distance in the target space.

The most significant difference in the calculation process between Isomap and MDS is the calculation of distance matrix. MDS calculates the distance matrix of the data in a high-dimensional space based on the Euclidean distance, while Isomap calculates the distance matrix based on the geodesic distance approximation. The geodesic distance is approximated as the shortest path between two points along the nonlinear manifold surface.

**Fig. 1:**
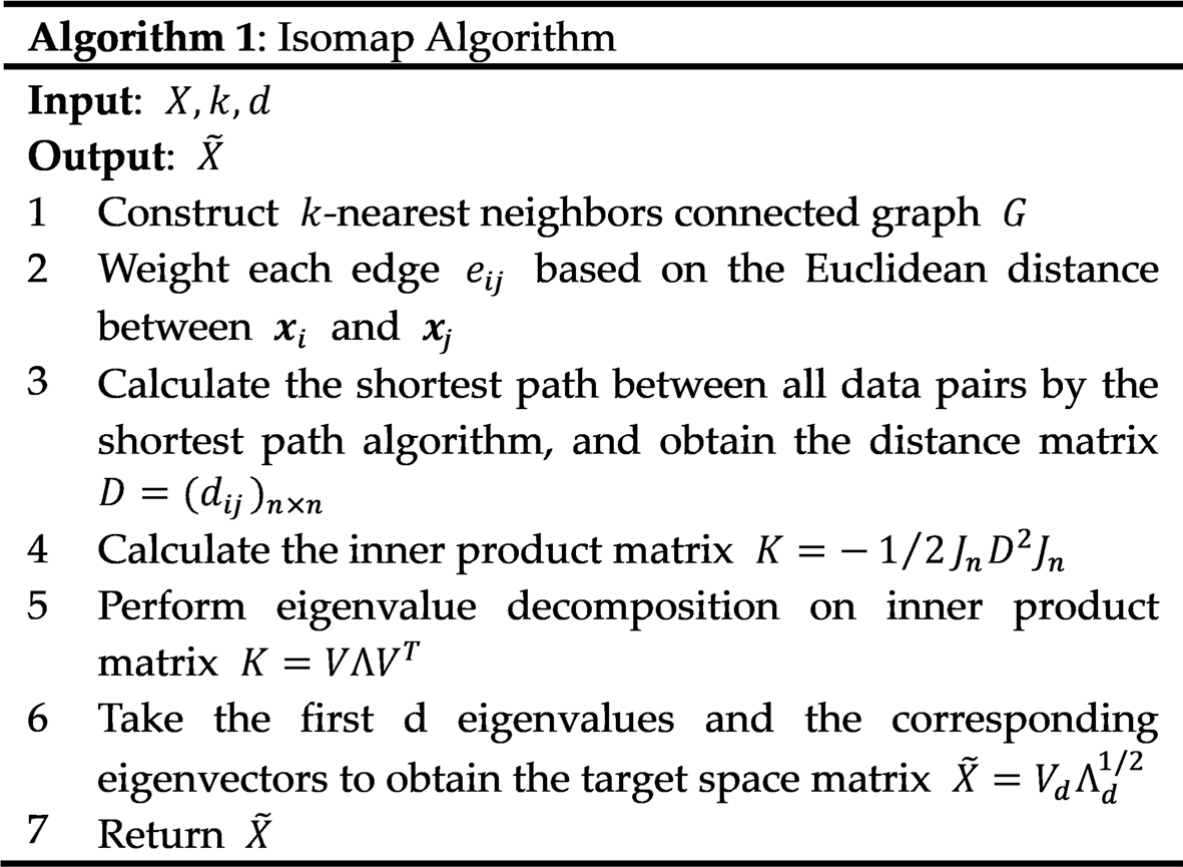
Algorithm 1: Isomap Algorithm

The pseudo-code of the Isomap algorithm can be presented in Fig. 1.

An Isomap process can be defined as:

1 $$\widetilde{X}={Isomap}_{p\to d}\left(X\right),$$

where $X$ is the original high-dimensional data, $X$ includes $n$ samples in ${\mathbb{R}}^{p}$, while $\widetilde{X}$ is the low-dimensional data in the target space ${\mathbb{R}}^{d} (d<p)$.

Two parameters need to be determined, including *k* in the k-nearest neighborhood graph and *d*, which is the dimensionality of the target space.

First, for each data point, the nearest $k$ points are connected by edges to construct a neighborhood graph $G$. The weight of each edge ${e}_{ij}$ is the Euclidean distance $|{{\varvec{x}}}_{i}-{{\varvec{x}}}_{j}|$, $i,j=1, \dots , n$.

Then, the geodesic distance between each pair is estimated by determining the shortest path in the neighborhood graph $G$. Here, the Warshall–Floyd algorithm is adopted to search for the shortest path. After this step, the estimated geodesic distance matrix $D={({d}_{ij})}_{n\times n}$ contains the shortest path distances between all pairs of data points. To ensure the symmetry of the distance matrix $D$, if there is a case where one point is the nearest neighbor of another point while the latter is not the nearest neighbor of the former, then the former would be connected to the latter [39].

The following steps are the same as those used in the classical MDS. The inner product matrix can be calculated as:

2 $$K=-1/2\,{J}_{n}{D}^{2}{J}_{n},$$

where ${J}_{n}={I}_{n}-1/n{1}_{n}{1}_{n}^{T}$, ${D}^{2}={({d}_{ij}^{2})}_{n\times n}$, ${I}_{n}=diag(\mathrm{1,1},\dots ,1)$ is the identity matrix of size *n*, and ${1}_{n}=(\mathrm{1,1},\dots ,1)$ is the 1-vector of size *n*.

Next, we conduct the eigenvalue decomposition on $K$ to obtain the eigenvector $V$ and eigenvalue matrix $\Lambda$:

3 $$K=V\Lambda {V}^{T},$$

For the determined target dimensionality $d$, we take the first $d$ eigenvalues and corresponding eigenvectors to calculate the coordinate matrix $\widetilde{X}$ of the target space ${\mathbb{R}}^{d}$.

### Proposed Isomap-embedded GA (Iso-GA) method

**Fig. 2:**
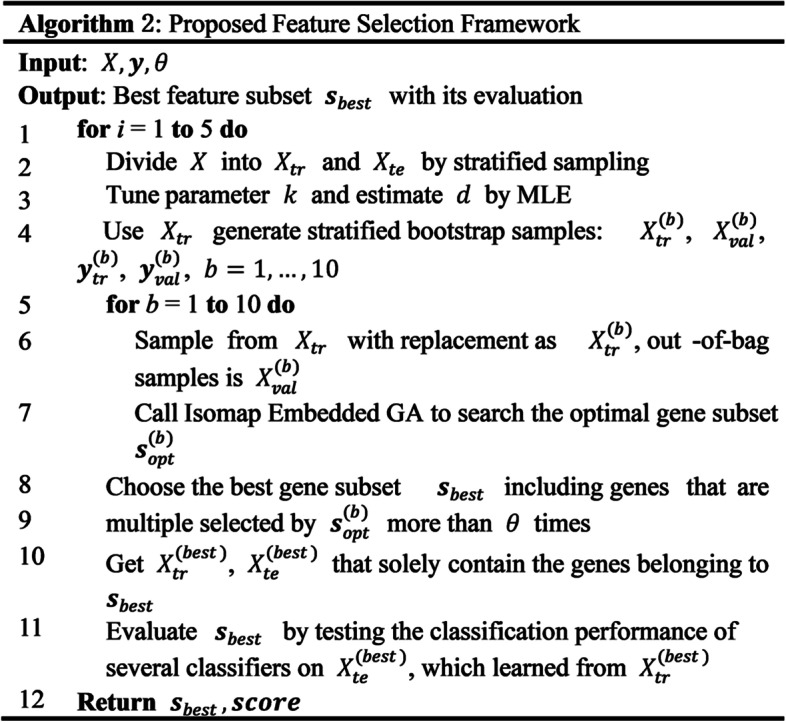
Algorithm 2: proposed feature selection framework

**Fig. 3:**
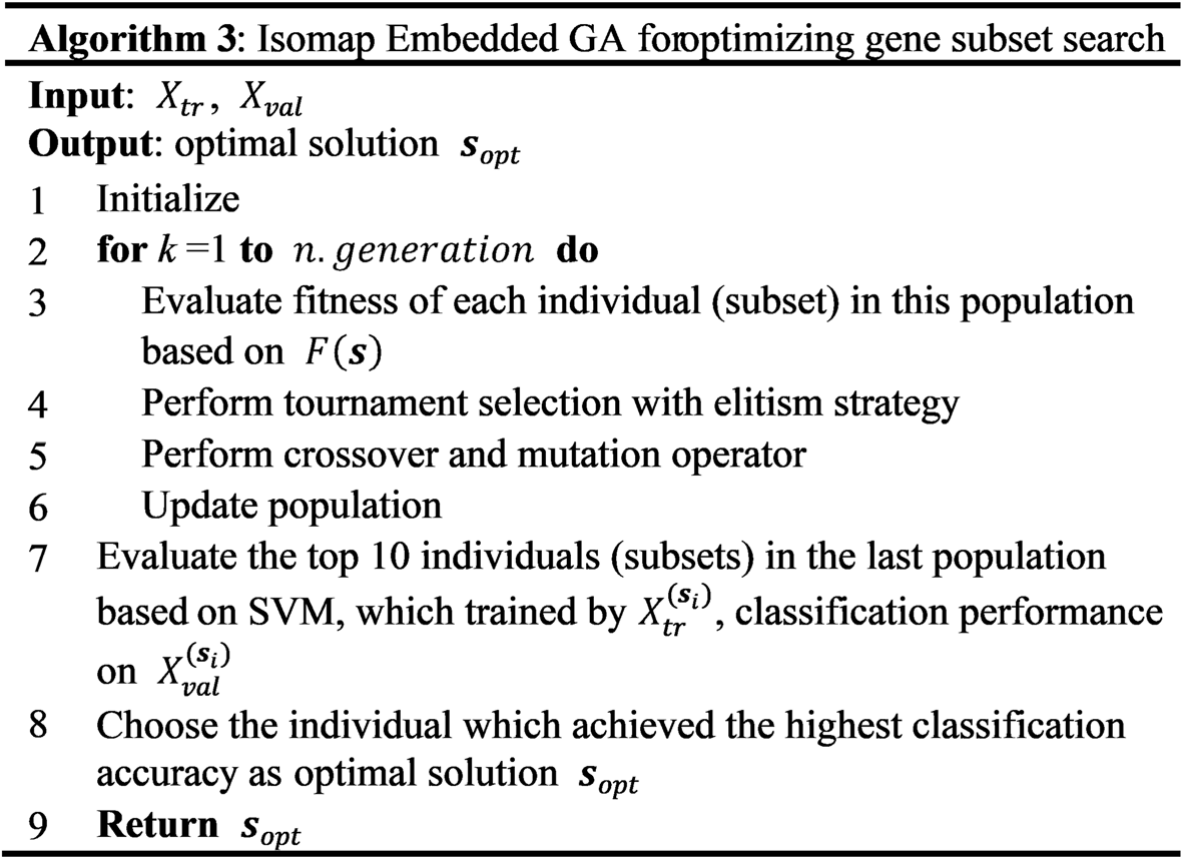
Algorithm 3: Isomap embedded GA for optimizing gene subset search

**Fig. 4:**
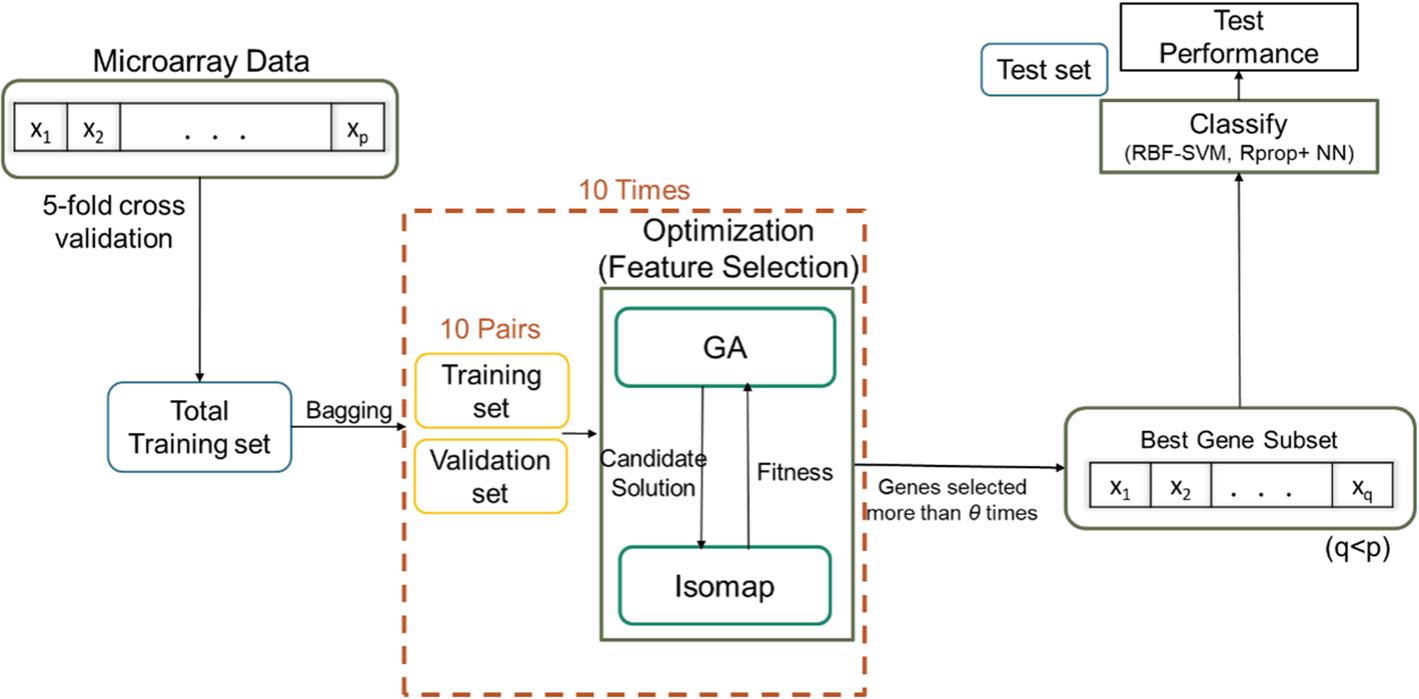
Framework flowchart of the proposed feature selection method

The pseudo-code of our feature selection framework and Iso-GA are presented in Figs. 2 and 3. A flowchart of our proposed feature selection framework is illustrated in Fig. 4.

The basic idea of GA is to imitate the natural selection process, where individuals with high fitness survive, while those with low fitness are eliminated. After several generations, the individual with the highest fitness is finally obtained, which represents the optimal solution to the challenge of interest. Therefore, the fitness value for optimization in the GA is a key parameter, whose choice is related to the judgement of the feature subset.

Here, all candidate feature subsets are binary-coded for each individual, where “1” and “0” denote that the feature corresponding to the location is selected and excluded, respectively. Based on the results of prior testing, we set the number of features selected by each individual to 30, i.e., each contains solely 30 bytes of “1”.

We define the fitness function as the DB Index of ${\widetilde{X}}^{(s)}$:

4 $$F\left({\varvec{s}}\right)=DB\left({\widetilde{X}}^{\left({\varvec{s}}\right)}, {\varvec{y}}\right),$$

5 $${\widetilde{X}}^{({\varvec{s}})}={Isomap}_{p\to d}\left({X}^{\left({\varvec{s}}\right)}\right),$$

where ${X}^{(s)}$ is the subset of $X$ that solely includes features belonging to ${\varvec{s}}$, and ${\widetilde{X}}^{(s)}$ is the matrix after mapping from the *p*-dimensional to the *d*-dimensional space by the Isomap algorithm.

The DB index is based on the following ideas: an accurate classification should have high inter-class and low intra-class dispersions; that is, the ratio of intra-class dispersion to inter-class dispersion should be small. As such, the smaller the DB index, the clearer the division of the data. Thus, the optimal solution with the smallest DB index is the feature subset for which each data class can be most clearly partitioned after the Isomap dimensionality reduction.

We assume that clearer partitioning means that the contained features contribute more to the classification. Thus, more accurate classification results can be obtained. To verify this assumption, we performed simulation experiments using a support vector machines (SVM) classifier. We randomly selected 500 random feature subsets of size 30 for each dataset (A detailed description of the datasets is provided in Datasets and Preprocessing). The DB index of all random subsets was calculated after the dimensionality reduction using Isomap. The micro-AUC (introduced in Evaluation Metrics) value of the test set was calculated, and scatter plots of the results are plotted in Additional file 1: Fig. S1.

The majority of the datasets indicate a negative correlation; however, the data points are sparsely scattered on both sides of the regression line. This result suggests that even if the DB Index is minimal, it does not necessarily mean that the classification performance is the best; however, if we directly search for the feature subset with the highest accuracy, it will be very time-consuming. Therefore, it is a reasonable and feasible solution to considerably narrow down the search scope by determining the smallest DB index.

After each GA search is completed, solely the SVM prediction accuracy of the validation set of the best 10 individuals in the last generation is calculated, and the individual with the highest accuracy is selected as the optimal solution ${{\varvec{s}}}_{opt}$ for this GA search.

Owing to the random GA search process, not all optimal subset genes obtained in each search are relational and informative for cancer classification. To obtain the genes that are not randomly selected, we set a threshold, $\theta$. If the number of selections in the 10 GA searches is higher than $\theta$, it will be included in the best gene subset ${{\varvec{s}}}_{best}$.

Finally, we adopted the classifiers to evaluate the obtained best gene subset ${{\varvec{s}}}_{best}$.

Regarding the classifier selection, SVM has demonstrated a better performance than the other existing machine learning algorithms in current research on two-class and multiclass microarray classification problems [40]. The features of SVMs include flexibility in the choice of similarity functions, the ability to handle data with large feature spaces, and the ability to obtain sparse solutions, making them suitable for gene expression data analysis [41]. Therefore, we chose the SVM as one of the major classifiers in this study.

The artificial neural network is an algorithm that simulates the structure and activity of neurons in the human brain. It comprises a series of neurons and connected layers. Backpropagation (BP) is the most popular algorithm for training a neural network by adjusting the synaptic weights [32].

The radial basis function kernel support vector machine (RBF-SVM) and resilient backpropagation with a weight backtracking neural network (Rprop + NN) are used as classifiers to evaluate the performance of the selected feature subsets.

As illustrated in Fig. 4, a fivefold cross-validation test was performed. The entire training set ${X}_{tr}$ is adopted for parameter tuning and feature selection, as well as for the learning process of classifiers, and the test set is used to test the accuracy of the classification results. The details of the cross-validation test are described in *Nested Cross-Validation*.

We use the kofnGA package [42] and RDRToolbox package [43] in R to implement the genetic algorithm feature selection and Isomap algorithm, respectively. All the experiments are performed in the R environment.

### Parameter selection and tuning

In the calculation process, the hyperparameters *d* and* k* are required as the input to the Isomap algorithm.

The parameter *d* is the dimensionality of the target space, which should be equal to the potential intrinsic dimensionality of data in the ideal case; however, the intrinsic dimensionality depends on the dataset and is difficult to determine in advance. The maximum likelihood dimensions estimator (MLDE) [44] method is used to automatically determine the dimensions of the target space of the Isomap algorithm.

For the parameter *k* of Isomap, we optimized the value of* k* using a grid search with a search range of [5, 20]. The* k* with the smallest DB index value after the Isomap dimensionality reduction is regarded as the optimal value.

After the parameter tuning with the entire training set ${X}_{tr}$, 10 pairs of training and validation sets were randomly generated by the bootstrap bagging method. For each training set ${X}_{tr}^{\left(b\right)}$, an Iso-GA was performed, and finally, 10 optimal gene subsets were obtained.

To determine the threshold *θ*, we calculated the probability of being selected at random to be less than 5%, depending on the size of different datasets.

For simplicity of calculation, if the selection of gene subsets is random, we assume that all genes will be selected with the same probability. Each GA search can be regarded as a Bernoulli trial, and the probability of being selected in each trial can be calculated as $p=v/n.gene$ (where $v$ is the size of the optimal subset and $n.gene$ is the number of genes). Then, the number of times selected in 10 GA runs ($X$) follows the Binomial distribution: $X\sim B(10,p)$. The probability of a gene being selected θ times is:

6 $$P\left(X=\theta \right)=C(\theta , 10){p}^{\theta }{(1-p)}^{10-\theta }$$

According to the number of genes in the dataset, we calculated the minimum $\theta$ value that can make the probability $\sum_{k=0}^{\theta }P\left(X=k\right)$ more than 0.95 as the threshold to obtain the best gene subset. This ensures that a gene selected more than *θ* times owing to randomness is a small probability event with a probability of less than 5%. Here, we consider that this gene is not selected randomly but correlates with cancer classification.

We applied the grid search method to optimize the parameters of each classifier. The parameter tuning ranges of RBF-SVM and Rprop + NN are provided in Tables 1 and 2, respectively.

**Table 1:** Parameter tuning range of RBF-SVM

RBF-SVM parameters	Tuning range
Sigma (kernel width)	[0.001, 0.011, 0.021, …, 0.091]
C (cost of constraints violation)	[1, 2, 3, …, 10]

**Table 2:** Parameter tuning range of Rprop + NN

Rprop + NN parameters	Tuning range
$hn1$	[10, 12, 14, …, 30]
$hn2$	[4, 6, 8, …, 20]

$hn1$: Number of nodes in the 1st hidden layer

$hn2$: Number of nodes in the 2nd hidden layer

For each parameter combination, we performed two threefold cross-validations to measure the average prediction accuracy.

### Computational complexity analysis

The proposed model is a hybrid method, and we discuss the computational complexity separately for each algorithm used in it. We can determine the complexity of MLDE, DB-Index, and Isomap based on previous studies [34, 45–47]. As a result, the computational complexity of the proposed Iso-GA is $O\left({n}^{3}\right)$ and the complexity of parameter selection for Isomap is $O\left(\mathrm{log}n\right)+O(p)$ [See a more detailed explanation in Additional file 1].

The computational complexity of the two classifiers used in this study is not discussed here, as they are not part of our proposed Iso-GA method and can be substituted with other classifiers.

### Datasets and preprocessing

This study used eight benchmark cancer microarray datasets to evaluate the performance of the proposed method. We used the datasets processed by Zhu et al. [40], and these datasets are originally published in literature [48, 49]. We presented a summary in Table 3. These datasets include cancer types such as breast, central nervous system, colon, leukemia, lung, lymphoid, and small round blue cell tumor. The number of features ranges from over 24,000 to only 2,000, and the target variables include both binary and multiclass classification situations, ranging from prognostic status to cancer subtype classification.

**Table 3:** Overview of benchmark microarray datasets

Dataset	Cancer type	No. of total genes	No. of total samples	Class (no. of samples)	Classification type
Breast	Breast cancer	24,188	97	Relapse (46) Non-relapse (51)	Cancer subtypes
CNS	Central nervous system embryonal tumor	7129	60	Survivors (39) Failures (21)	Prognosis
Colon	Colon cancer	2000	62	Tumor (40) Normal (22)	Cancer and Normal
Leukemia	Human acute leukemias	7129	72	ALL (47) AML (25)	Cancer subtypes
Lung	Lung cancer	12,600	203	ADEN (139) SCLC (6) SQUA (21) COID (20) Normal (17)	Cancer subtypes and normal
Lymphoma	Adult lymphoid malignancies	1230	66	DLBCL (46) FL (9) CLL (11)	Cancer subtypes
MLL	Mixed-lineage leukemia	12,582	72	ALL (24) MLL (20) AML (28)	Cancer subtypes
SRBCT	Small,​ round blue cell tumors of childhood	2308	83	EWS (29) BL (11) NB (18) RMS (25)	Cancer subtypes

The Lymphoma dataset contains several missing data. The genes with missing values were removed. In addition, a few genes in the Breast and Lymphoma datasets had the same expression values. Such genes are meaningless for classification prediction. Therefore, they were removed directly. A statistical summary of the final datasets after removal is provided in Table 3.

Because the various gene expressions in the datasets can affect the classification performance, the datasets were standardized. The samples containing several outliers were removed.

Owing to several irrelevant and redundant features in the microarray data [40], the GA search space becomes vast, thereby decreasing search efficiency and computational speed. Although GA has good global search performance, the existence of several redundant features significantly increases the randomness of the GA search.

Therefore, we calculated the information gain between the target variable and each gene. Information gain is a measure based on entropy, higher information gain means a higher correlation between feature and classification [50]. We determined that the information gain of a vast number of genes was 0. This means that different classification labels do not increase the amount of information on these genes. Therefore, we removed these genes from the preprocessed datasets. The gene numbers after removal and the thresholds* θ* for the best gene subset selection for each dataset are presented in Table 4.

**Table 4:** Gene numbers of each dataset after preprocess and ${\varvec{\theta}}$ value

Dataset	No. of genes	$\theta$
Breast	982	2
CNS	70	8
Colon	136	5
Leukemia	996	2
Lung	9564	2
Lymphoma	2153	2
MLL	5194	2
SRBCT	668	3

### Evaluation methods

#### Evaluation metrics

The accuracy ($Acc$) is commonly adopted as the classifier evaluation index for classification problems, and the formula of $Acc$ is formulated as follows:

7 $$Acc=(TP+TN)/(P+N),$$

where *P* and *N* are the numbers of positive and negative samples, respectively, while *TP* and *TN* denote the numbers of positive and negative samples that were correctly predicted by the classifier.

One disadvantage of $Acc$ is that it depends on the choice of the classification threshold when the output of the classifier is the probability of each class. The area under the receiver-operating characteristic curve (AUC), which is not affected by the threshold, is a better choice.

In this study, however, there are multiple labels in the datasets to which AUC is not available. Therefore, all the performance metrics, including the average accuracy indices, macro-AUC, and micro-AUC, were used to evaluate the classifier performance. The macro approach averaged the values of metric M for each class, while the micro approach aggregated the values of all contingency tables for each class and then computed the metric M interested across all classes [51]:

8 $${M}_{macro}=\frac{1}{C}\sum_{i=1}^{C}M({tp}_{i}, {fp}_{i}, {tn}_{i}, {fn}_{i}),$$

9 $${M}_{micro}=M\left(\sum_{i=1}^{C}{tp}_{i}, \sum_{i=1}^{C}{fp}_{i}, \sum_{i=1}^{C}{tn}_{i}, \sum_{i=1}^{C}{fn}_{i}\right).$$

Here, metric M is the AUC. As there is no consensus about macro- and micro- approaches [51], both metrics are considered in this study.

#### Nested cross-validation

**Fig. 5:**
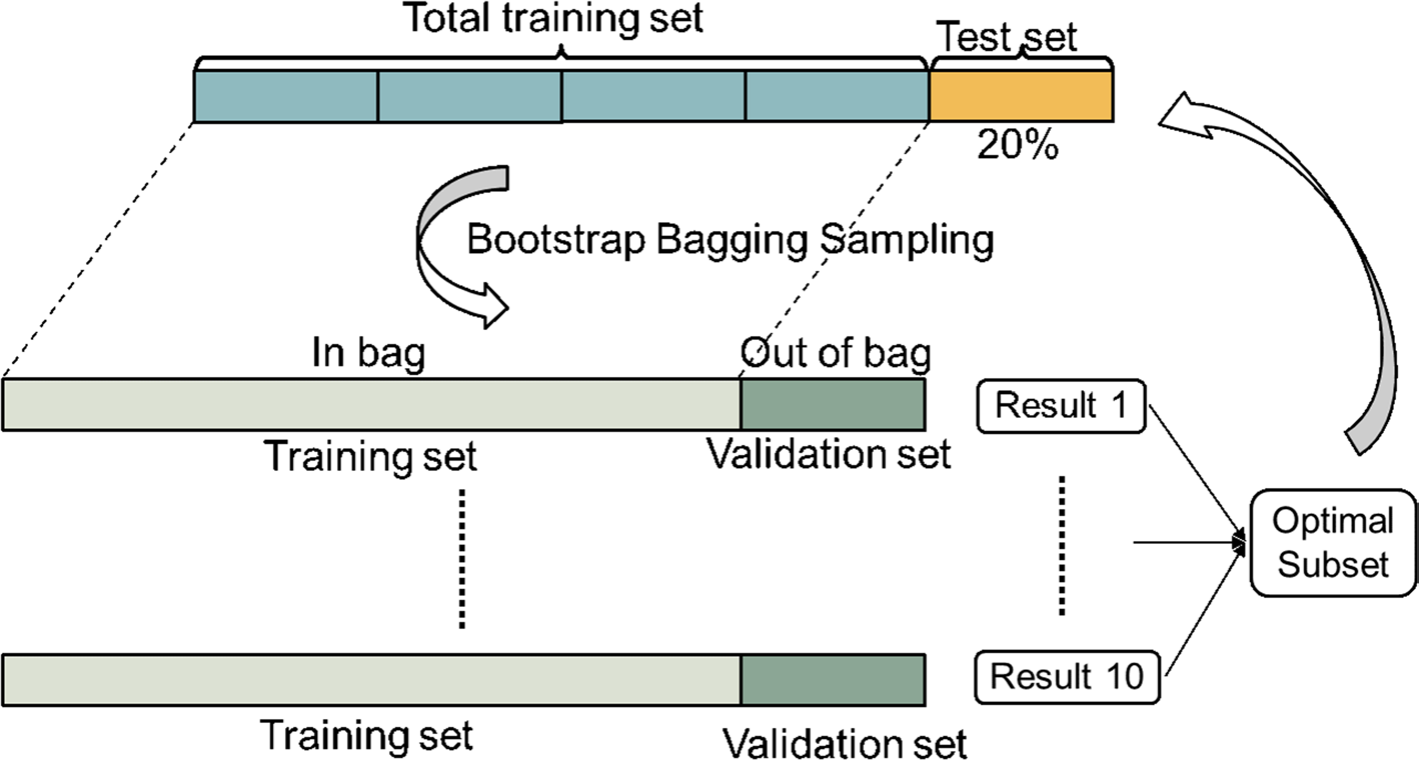
Nested cross-validation method in the proposed method

In this study, the nested cross-validation method was adopted, in which the outer and inner sides were cross-validated separately (Fig. 5).

For the entire dataset, we used a stratified sampling method to divide it into five folds. One was used as a test set, while the remaining four folds were used as the training set. The sample proportions of different classes in each fold were consistent with those of the population.

Simultaneously, validation is necessary for the inner loop (i.e., parameter tuning). Because the number of samples was small, we adopted the stratified bootstrap aggregating (bagging) method to explore the optimal feature subset on the inner side for the GA search process.

The stratified bagging method uses random sampling with replacement to sample each class of data separately. Then, the sampling results of all classes are combined to generate the in-bag set. The out-of-bag sample, which is not selected, is used as the validation set, and there is no duplication. We set the bootstrap sample size to be the same as the original dataset (i.e., the entire training set) and sampled it 10 times. The random simulation results indicate that all samples can be selected into the training set at least once after 10 bagging sampling.

#### Ranking score

Feature selection aims to build a higher-accuracy model with fewer features. When there is no significant difference in accuracy, we tend to consider that using fewer features is better. Therefore, we adopted the ranking score $\mathcal{R}$ here to compare and evaluate the feature selection methods comprehensively.

We calculated the ranking of each model for each metric. The higher the classification performance, the higher the ranking; simultaneously, the fewer the number of features selected, the higher the ranking. The ranking score $\mathcal{R}$ is defined as follows:

10 $$\mathcal{R}=\frac{\sum_{j=1}^{{N}_{model}}\sum_{i=1}^{{N}_{metric}}{r}_{i}^{j}}{{N}_{model}{\cdot N}_{metric}}+{r}_{|\mathbf{s}|},$$

where ${r}_{i}^{j}$ denotes the sum of rankings of the ith metric of the jth model on all datasets, while ${r}_{|\mathbf{s}|}$ denotes the sum of rankings of $|\mathbf{s}|$ for all datasets, respectively.

#### Model comparison

We compare different feature selection methods from two aspects. First, to verify the effectiveness of the Isomap algorithm in our proposed framework, the MDS-embedded GA (MDS-GA) method and GA method without any dimensionality reduction were also conducted. All methods used the same hierarchical fivefold cross-validation training and test sets to ensure fair comparisons.

We then considered the CER-ABC feature selection process proposed by Beatriz et al. [32] and the Markov-embedded genetic algorithm (MBEGA) for gene selection proposed by Zhu et al. [40] as competitive models. These two methods have shown promising performance in the gene selection of microarray data.

Concisely the CER-ABC method used the artificial bee colony (ABC) algorithm, one of the most popular metaheuristic algorithms such as the genetic algorithm [10], as an optimization technique, and the classification error function (CER) was used as the fitness function. We use the metaheuristicOpt [52] package in R to implement the ABC algorithm. The parameters of ABC are set according to the reported parameters in the original paper, and default parameters are applied for unreported ones. For the threshold $th$, i.e., the probability that a gene can be selected, we used the one that achieved the highest accuracy for each dataset to obtain the feature subset as the result of this method. Because our goal is to compare the effectiveness of feature selection, we only utilized the feature selection results of CER-ABC and assessed them using the same classifiers and tuning approach as Iso-GA.

The MBEGA method [40] is similar to our proposed method, which is also a GA-based gene selection method. We compared our method with MBEGA and used the same datasets as those used in developing MBEGA. We relied on the published results of MBEGA for comparison without implementing this method ourselves.

## Results

### Results based on the proposed framework

As described above, we first verified the effectiveness of the Isomap algorithm within the same framework. The subset of the entire training set that solely contains the genes selected by the feature selection method is used to train the classifier, while the test set is used to evaluate the performance of the trained classifier.

Accordingly, three models were tested, i.e., Iso-GA, MDS-GA, and GA. All models follow the proposed framework. The following part presents the performance of RBF-SVM and Rprop + NN trained on the feature subsets selected by each method on each dataset, including two evaluation indicators, Macro-AUC and Micro-AUC.

Tables 5 and 6 present the average macro- and micro-AUC values of the RBF-SVM classifier and their corresponding standard deviations in the outer fivefold cross-validation. The best results obtained for each dataset are indicated in bold.

**Table 5:** Macro-AUC means (standardized variance) of RBF-SVM classification on gene subsets selected by feature selection algorithms

Dataset	Iso-GA	MDS-GA	GA
Breast	**0.857 (0.070)**	0.837 (0.07)	0.825 (0.086)
CNS	0.710 (0.092)	**0.796 (0.052)**	0.793 (0.052)
Colon	0.874 (0.120)	**0.884 (0.067)**	0.826 (0.097)
Leukemia	**0.962 (0.001)**	0.954 (0.016)	0.939 (0.051)
Lung	0.956 (0.018)	**0.965 (0.016)**	0.943 (0.014)
Lymphoma	**0.964 (0.004)**	**0.964 (0.004)**	0.963 (0.003)
MLL	0.956 (0.034)	0.957 (0.034)	**0.965 (0.019)**
SRBCT	**0.978 (0.003)**	**0.978 (0.003)**	**0.978 (0.004)**

**Table 6:** Micro-AUC means (standardized variance) of RBF-SVM classification on gene subsets selected by feature selection algorithms

Dataset	Iso-GA	MDS-GA	GA
Breast	**0.869 (0.063)**	0.838 (0.092)	0.824 (0.098)
CNS	0.763 (0.086)	**0.820 (0.047)**	0.814 (0.054)
Colon	0.900 (0.086)	**0.907 (0.076)**	0.849 (0.100)
Leukemia	**1.000 (0.000)**	0.985 (0.032)	0.976 (0.055)
Lung	0.975 (0.007)	**0.981 (0.009)**	0.971 (0.005)
Lymphoma	**1.000 (0.000)**	0.999 (0.001)	0.998 (0.003)
MLL	0.983 (0.025)	0.980 (0.034)	**0.989 (0.024)**
SRBCT	0.996 (0.009)	**0.997 (0.006)**	0.993 (0.007)

The proposed Iso-GA achieved the highest average macro- and micro-AUC values for the Breast, Leukemia, and Lymphoma datasets. The average value of the micro-AUC of Leukemia and Lymphoma was 1, and the standard deviation was 0.

The highest average macro- and micro-AUC values were attained using MDS-GA for the CNS, Colon, and Lung datasets. All three methods showed similar performances on the SRBCT dataset.

We performed the Wilcoxon signed-rank test to test the statistical significance of the results of the five folds obtained by the different methods. The result of Iso-GA was used as a benchmark to test whether MDS-GA and GA were statistically significantly different from it. We also calculated the ranking of each method in terms of macro- and micro-AUC (indicated as Ma-rank and Mi-rank, respectively). These results can be found in Additional file 1: Table S1.

Based on the Wilcoxon signed-rank test results, we found that most of the differences among the three methods were not statistically significant. According to the sum of rankings, the sum of the AUC rankings of the MDS-GA method for the two classifiers is higher. Therefore, in the proposed GA-based feature selection framework, the subset of genes selected by the Iso-GA method had a slightly lower classification performance than MDS-GA on the RBF-SVM classifier.

Similarly, Tables 7 and 8 present the average macro- and micro-AUC values of the Rprop + NN classifier and their corresponding standard deviations in the outer fivefold cross-validation. The best results obtained for each dataset are indicated in bold.

**Table 7:** Macro-AUC Means (standardized variance) of Rprop + NN Classification on Gene Subsets Selected by Feature Selection Algorithms

Dataset	Iso-GA	MDS-GA	GA
Breast	0.792 (0.028)	0.781 (0.135)	**0.795 (0.054)**
CNS	0.644 (0.161)	**0.778 (0.103)**	0.680 (0.064)
Colon	**0.867 (0.109)**	0.789 (0.136)	0.834 (0.087)
Leukemia	**0.955 (0.010)**	0.944 (0.025)	0.926 (0.080)
Lung	**0.959 (0.011)**	0.956 (0.031)	0.949 (0.028)
Lymphoma	0.929 (0.063)	**0.964 (0.004)**	**0.964 (0.004)**
MLL	**0.953 (0.036)**	0.918 (0.062)	0.946 (0.046)
SRBCT	0.966 (0.020)	**0.968 (0.027)**	0.927 (0.087)

**Table 8:** Micro-AUC means (standardized variance) of Rprop + NN classification on gene subsets selected by feature selection algorithms

Dataset	Iso-GA	MDS-GA	GA
Breast	**0.807 (0.019)**	0.803 (0.133)	0.788 (0.058)
CNS	0.679 (0.166)	**0.810 (0.051)**	0.732 (0.031)
Colon	**0.885 (0.102)**	0.828 (0.102)	0.834 (0.087)
Leukemia	**0.987 (0.018)**	0.977 (0.027)	0.965 (0.075)
Lung	0.958 (0.020)	**0.974 (0.024)**	0.949 (0.028)
Lymphoma	0.975 (0.033)	**0.997 (0.005)**	**0.997 (0.005)**
MLL	**0.973 (0.038)**	0.936 (0.067)	0.971 (0.049)
SRBCT	0.991 (0.008)	**0.997 (0.004)**	0.993 (0.013)

The subset of genes selected by the Iso-GA method, according to the macro-AUC values of Rprop + NN classifier on classification, outperformed the other two methods on the five datasets, including Breast, Colon, Leukemia, Lung, and MLL.

Based on the Micro-AUC values, the performance on the five datasets, Breast, Colon, Leukemia, and MLL, was better than that of the other two methods. In addition, although not the highest, the results for Lung and SRBCT datasets, 0.975 and 0.991, respectively, can be considered very close to the optimal results of 0.974 and 0.997, with a marginal difference.

Similarly, the ranking of the performance of each method and the *p* value of the Wilcoxon sign rank test on the different datasets are shown in Additional file 1: Table S2.

According to the Wilcoxon signed-rank test results, most of the differences between the three methods were not statistically significant; however, the Iso-GA method had the highest sum of rankings in the overall AUC rankings for the two classifiers.

Overall, in the proposed GA-based feature selection framework, the Rprop + NN classifier obtained from the subset of genes selected by the Iso-GA method outperformed the MDS-GA and GA methods.

Because the primary aim of feature selection is to reduce the data dimensionality, it is better to select fewer genes when there is no significant improvement in classification accuracy. The average number of genes selected by each method, $|s|$, is summarized in Table 9. The optimal result obtained on each dataset, i.e., the minimum average size, is shown in bold.

**Table 9:** Average size of selected gene subset by each method

Dataset	Proposed feature selection Framework			CER-ABC	MBEGA
	Iso-GA	MDS-GA	GA		
Breast	51.4 (4.5)	53.8 (3.3)	56.4 (3.8)	127.4 (8.3)	**14.5 (4.2)**
CNS	**4.0 (1.4)**	7.0 (1.4)	8.2 (0.8)	18.4 (2.9)	20.5 (6.9)
Colon	**11.8 (2.5)**	17 (2.0)	18.4 (2.7)	19.6 (4.0)	24.5 (7.0)
Leukemia	43.2 (3.3)	52.4 (3.0)	54 (4.9)	144.2 (18.3)	**12.8 (4.9)**
Lung	18 (8.0)	32.4 (4.9)	37.2 (5.3)	998.6 (30.02)	**14.1 (7.0)**
Lymphoma	48.4 (3.7)	66.6 (4.5)	71.2 (1.1)	112 (7.7)	**34.3 (8)**
MLL	**21 (4.1)**	31.8 (5.6)	38.2 (7.9)	1538.4 (56.0)	32.1 (10.6)
SRBCT	**17.6 (1.9)**	30.4 (1.8)	32 (2.9)	568.2 (8.3)	60.7 (11.7)

The ranking score $\mathcal{R}$ of the classification performance of the two classifiers and the ranking of the selected feature subset sizes of these three methods are provided in Table 10.

**Table 10:** Ranking summation

	Iso-GA	MDS-GA	GA
RBF-SVM Ranking	27.8	23.8	39.3
Rprop + NN Ranking	29	31	36
|**s**| Ranking	8	16	24
Ranking Score	64.8	70.8	99.3

${\overline{r} }^{(RBF-SVM)}$: The sum of average ranking of RBF-SVM on all datasets

${\overline{r} }^{(Rprop+NN)}$: The sum of average ranking of Rprop + NN on all datasets

In summary, the proposed Iso-GA method achieved the best overall performance ($\mathcal{R}=22.2$), indicating that it can select fewer genes while achieving a high classification accuracy.

### Comparison with other existing methods

Because the performance metric adopted in these comparison models is the average classification accuracy, and we did not have the codes of MBEGA to calculate its macro- and micro-AUC, we compared the results based on the average accuracy.

The Wilcoxon signed-rank tests were performed to compare the accuracy results from the outer fivefold cross-validation of models in each dataset (Table 11). We performed separate tests depending on the classifier. Taking Iso-GA as a reference, if the average accuracy of classification of Iso-GA is higher, a one-sided test is performed; if the *p* value is less than the given significant level, the result of Iso-GA is significantly higher than that of the compared method, otherwise, no significant difference is indicated; if the average accuracy of classification of Iso-GA is lower, a two-sided test is performed, and if the *p* value is less than the given significant level, the result of Iso-GA is significantly different from the compared method, otherwise it means there is no significant difference. “**” denotes a significance level of 0.05, and “*” denotes a significance level of 0.1.

**Table 11:** Average Accuracy of Each Methods with Results of Wilcoxon Signed-Rank Test

Dataset	Proposed feature selection framework						CER-ABC (SVM)	CER-ABC (NN)	MBEGA
	Iso-GA (SVM)	Iso-GA (NN)	MDS-GA (SVM)	MDS-GA (NN)	GA (SVM)	GA (NN)			
Breast	**0.821 ** ^Ref1^** (0.069)**	0.735 ^Ref2^ (0.080)	0.726 ^1 **^ (0.096)	0.769 ^2 ns^ (0.125)	0.748 ^1 **^ (0.109)	0.685 ^2 ns^ (0.068)	0.811 ^1 ns^ (0.058)	0.739 ^2 ns^ (0.075)	0.807 ^NA^ (0.035)
CNS	0.717 ^Ref1^ (0.139)	0.633 ^Ref2^ (0.139)	0.717^1 ns^ (0.075)	0.750 ^2 ns^ (0.059)	0.733^1 ns^ (0.037)	0.750 ^2 ns^ (0.083)	**0.867** ^1 ns^** (0.112)**	0.767 ^2 ns^ (0.070)	0.722 ^NA^ (0.060)
Colon	0.826 ^Ref1^ (0.097)	0.842 ^Ref2^ (0.143)	**0.858**^1 ns^** (0.081)**	0.791 ^2 ns^ (0.071)	0.826^1 ns^ (0.113)	0.792 ^2 ns^ (0.086)	0.844 ^1 ns^ (0.142)	0.760 ^2 *^ (0.089)	0.857 ^NA^ (0.055)
Leukemia	**1.000 ** ^Ref1^** (0.000)**	0.943 ^Ref2^ (0.060)	0.958^1 ns^ (0.063)	0.930 ^2 ns^ (0.051)	0.971^1 ns^ (0.064)	0.971 ^2 ns^ (0.064)	0.971 ^1 ns^ (0.064)	0.971 ^2 ns^ (0.064)	0.959 ^NA^ (0.025)
Lung	0.943 ^Ref1^ (0.014)	0.935 ^Ref2^ (0.021)	0.943^1 ns^ (0.015)	0.951 ^2 ns^ (0.013)	0.935^1 ns^ (0.010)	0.937 ^2 ns^ (0.026)	0.939 ^1 ns^ (0.016)	0.921 ^2 ns^ (0.006)	**0.990** ^NA^** (0.009)**
Lymphoma	**1.000 ** ^Ref1^** (0.000)**	0.980 ^Ref2^ (0.027)	0.990^1 ns^ (0.023)	0.980 ^2 ns^ (0.027)	0.990^1 ns^ (0.021)	0.971 ^2 ns^ (0.043)	**1.000** ^1 NA^** (0.000)**	0.970 ^2 ns^ (0.045)	0.977 ^NA^ (0.028)
MLL	**0.953** ^Ref1^** (0.048)**	**0.953** ^Ref2^** (0.033)**	**0.953**^1 ns^** (0.058)**	0.888 ^2 ns^ (0.104)	0.971^1 ns^ (0.064)	0.962 ^2 ns^ (0.062)	**0.953** ^1 ns^** (0.058)**	0.925 ^2 ns^ (0.043)	0.943 ^NA^ (0.033)
SRBCT	**0.994** ^Ref1^** (0.013)**	0.970 ^Ref2^ (0.021)	0.988^1 *^ (0.026)	0.982 ^2 *^ (0.026)	0.969^1 ns^ (0.022)	0.988 ^2 ns^ (0.017)	0.988 ^1 ns^ (0.016)	0.939 ^2 **^ (0.066)	0.992 ^NA^ (0.012)

**p value of Wilcoxon signed-rank test is less than the significance level of 0.05

*p value of Wilcoxon signed-rank test is less than the significance level of 0.1

*ns* no significant

The results in Table 11 indicate that the proposed Iso-GA method can achieve the best average accuracy on the RBF-SVM classifier for the five datasets (Breast, Leukemia, Lymphoma, MLL, and SRBCT). The maximum average accuracy achieved on each dataset is shown in bold.

For the CNS dataset, the gene subset selected by the CER-ABC algorithm achieved the best prediction accuracy on the RBF-SVM classifier, and the Colon and Lung datasets and the MBEGA method achieved the highest accuracy; however, the optimal gene subsets of the CNS and Colon selected by the Iso-GA algorithm were the smallest. Solely for the Lung dataset, the MBEGA method selected the fewest genes while achieving the highest accuracy.

To comprehensively compare these models, the rankings of the average prediction accuracy ${\overline{r} }^{Acc}$ and selected gene subset sizes ${r}_{|{\varvec{s}}|}$ are summarized in Table 12. As the results of the MBEGA method are based solely on the SVM classifier, the results of the SVM are considered in calculating the average accuracy ranking.

**Table 12:** Ranking score

	Proposed feature selection framework			CER-ABC (SVM)	MBEGA
	Iso-GA (SVM)	MDS-GA (SVM)	GA (SVM)		
${\overline{r} }^{Acc}$	18.5	27	27	20.5	25
${r}_{|{\varvec{s}}|}$	12	20	29	38	21
$\mathcal{R}$	30.5	47	56	58.5	46

According to the results, the proposed Iso-GA method achieved the highest-ranking score ($\mathcal{R}$ = 30.5), representing the best classification performance and smallest gene subset simultaneously.

### Feature selection results and visualization

Visualizing the dimensionality-reduced dataset is intuitive to verify whether the selected feature subsets are related to cancer classification and compare the classification performance.

**Fig. 6:**
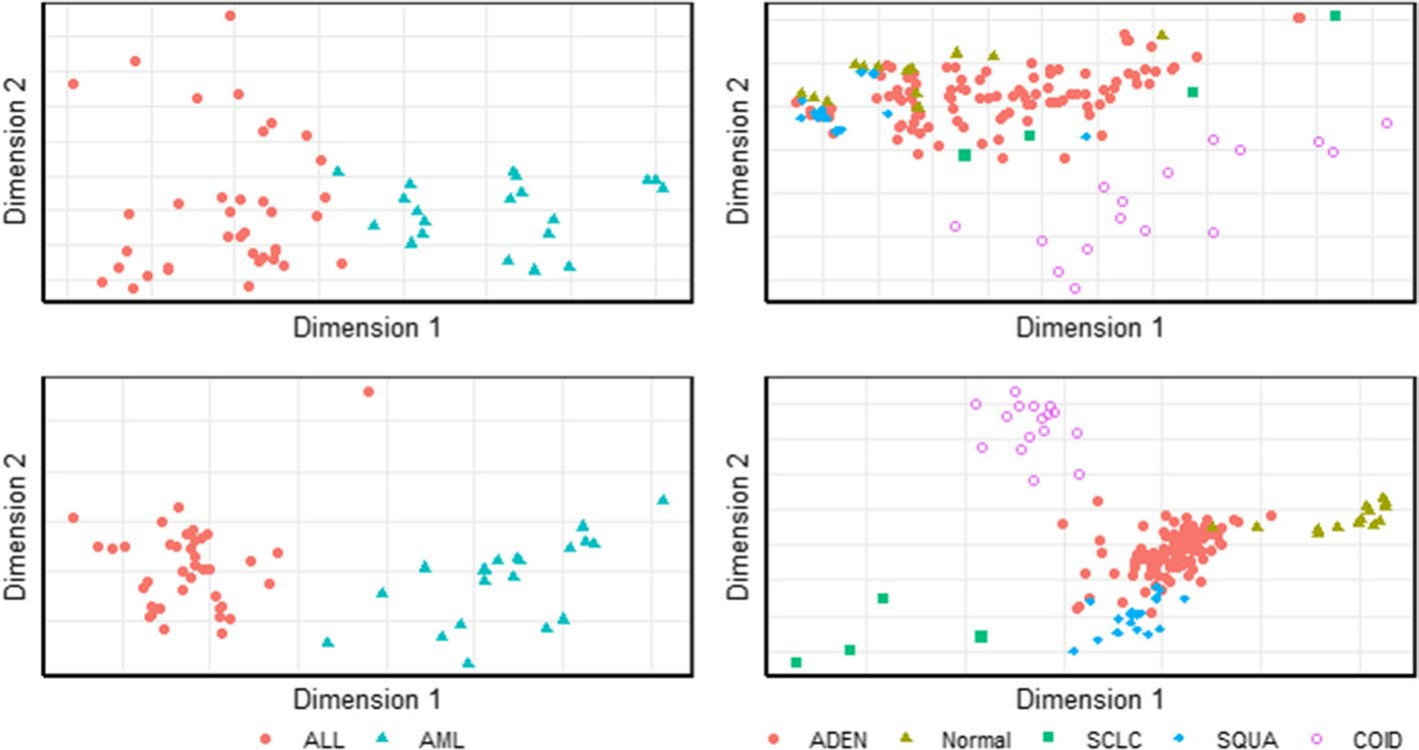
Visualization results of Leukemia (left) and Lung (right) dataset

We show the visualization results of two datasets, Leukemia and Lung (Fig. 6). The results of other datasets can be found in Additional file 1: Fig. S2. The upper panel illustrates the results of Isomap dimensionality reduction using all genes, and the lower panel illustrates the results of Isomap dimensionality reduction using solely the subset of genes selected by the Iso-GA method.

Even if all the genes are used, Isomap can obtain clearer results after dimensionality reduction, suggesting that our hypothesis that the microarray data are distributed on the nonlinear structure is reasonable.

Using the proposed Iso-GA selected genes, each class data point can be separated more clearly. This indicates that the proposed feature selection framework can effectively remove the noise and redundancy, which are irrelevant to classification, and as a result, it can obtain visible results that are easier to understand and explain.

## Discussions

In this study, we proposed a novel feature selection method called Iso-GA and a framework based on it. The proposed method could select a smaller subset of critical genes and improve the accuracy of cancer classification. The proposed method takes into account the nonlinear structure of gene expressions in microarray data and uses Isomap for dimensionality reduction and fitness evaluation. Moreover, the proposed framework reduced the randomness in the GA search algorithm by repeating the search process and selecting features based on a specified threshold. Thus, more noisy features could be removed with a limited number of the potentially cancer-related genes selected for cancer classification, and the overall accuracy of classifiers was improved. We found that Iso-GA exhibited efficient gene selection performance and achieved high accuracy in cancer classification. In addition, we also found that using nonlinear method might be a better choice for dimensionality reduction in microarray data.

The originality and significance of this study are summarized as follows:

1. This study innovatively hybrid the manifold learning algorithm Isomap with GA for feature selection. This hybridization takes into account the nonlinear structure of microarray data. Isomap maps the sample points distributed in non-Euclidean space to low-dimensional Euclidean space by calculating the geodesic distance between sample points. Comparison results showed that GA combined with Isomap achieved the highest-ranking score $\mathcal{R}$, indicating the best feature selection performance, compared to GA combined with the linear dimensionality reduction methods or without dimensionality reduction.
2. This study introduced an innovative approach to evaluating the correlation between feature subsets and cancer subtypes in GA. Instead of relying on classifier accuracy, we used the clarity of division between samples of different classes. The fitness of a solution in GA search is evaluated by the DB index, which avoids classifier dependency and can be applied easily to any other classifiers. The DB index enables inferences about the appropriateness of data partition and helps to assess which subset of genes can effectively partition gene features with different labels. However, as noted by Thomas et al. [53], the DB index evaluates the distance between clusters using Euclidean distance and does not consider the geometry of the spatial distribution of clusters. To address this limitation, Isomap is used in the proposed method to map the nonlinear microarray data to a low-dimensional linear space, considering the underlying geometry of the data distribution.
3. The proposed feature selection framework aims to mitigate the impact of algorithmic randomness in selecting features. Although the good global search performance of GA benefits from the random mutation, it can introduce randomness, leading to the selection of irrelevant features into the optimal subset of features. Therefore, we introduced a statistical method that calculates the outputs of multiple GA search results, and genes with a probability of less than 5% of being randomly selected are included in the optimal subset. The comparison results show that this improvement can select fewer genes while obtaining the same or even higher accuracy. This indicates that the proposed framework can potentially avoid the randomness of the metaheuristic algorithm.

The classification performance of the proposed method was compared to other existing ones on eight microarray datasets of different cancers. Iso-GA achieved the highest-ranking score $\mathcal{R}$, indicating that the highly accurate classification performance can be achieved by using a smaller gene subset size. Prior to applying the proposed models, these datasets were preprocessed by removing the missing values and outliers, and uninformative features were filtered out using information gain due to the presence of multiclass data sets that do not apply to the t-test. Iso-GA improves classification accuracy and preserves data interpretability. It has general applicability in that it can be extended to various classifiers. Although RBF-SVM and Rprop + NN were used in this study, Iso-GA could be combined with many other classifiers for cancer classification since the feature selection is independent of the classifiers.

However, there are several limitations. Firstly, we did not consider the factors of potential similarity and interaction among the genes, which may have some impact on the stability of the feature selection algorithm and the classification performance. Understanding these factors requires knowledge of biology and disease, which is beyond the scope of this study. Secondly, although Isomap is an effective method in various domains, it still has some shortcomings, such as topological instability and powerlessness in handling non-convex manifolds [54]. Isomap is an unsupervised dimensionality reduction technique, resulting in the incapability to use the class label information and embed new data points for testing or validation. Some extended Isomap-based methods have been proposed to solve this problem. For example, Multi-manifold Discriminant Isomap (MMD-Isomap) [55] and semi-supervised discriminant Isomap (SSD-Isomap) [56] may provide a better solution. Since the validation of Isomap is not necessary in our proposed framework, these extended methods are not considered here. Lastly, we assumed that gene microarray data are more likely to be in nonlinear space. However, the distribution of real-world gene expression data is far more complex, and it is difficult to verify the nonlinear space assumption. Nevertheless, the comparison results suggested that nonlinearity provides a better fit than linearity distance.

## Conclusions

In this study, we proposed a GA-based feature selection framework called Iso-GA to select the optimal subset of genes in microarray data. The framework embedded the Isomap algorithm for nonlinear dimensionality reduction to select genes that met a given probability-based threshold as the best for classification. Iso-GA exhibited efficient gene selection performance and achieved high accuracy in cancer classification.

## Supplementary Information

**Additional file 1.** This supplementary file includes a detailed description of the computational complexity analysis, as well as modeling details and results: (1) **Table S1**—The Rank of Macro-AUC and Micro-AUC of RBF-SVM Classification on Microarray Datasets and the P Value of Wilcoxon Sign Rank Test (2) **Table S2**—The Rank of Macro-AUC and Micro-AUC of Rprop+ NN Classification on Microarray Datasets and the P Value of Wilcoxon Sign Rank Test (3) **Table S3**—The parameter selection and tunning range (4) **Figure S1**—The regression fitting results of the classification accuracy of gene subsets with different DB values (5) **Figure S2**—Visualization results of each dataset.

## Data Availability

The microarray datasets analyzed in this study are available at http://csse.szu.edu.cn/staff/zhuzx/Datasets.html.
