# Supplementary material for: Genetic algorithm-based feature selection with manifold learning for cancer classification using microarray data
Source: BMC Bioinformatics. 2023 Apr 8;24:139. doi: 10.1186/s12859-023-05267-3 (PMC10082986; doi:10.1186/s12859-023-05267-3)
Supplement: Supplementary file 1 — Additional file 1. This supplementary file includes a detailed description of the computational complexity analysis, as well as modeling details and results: (1) Table S1—The Rank of Macro-AUC and Micro-AUC of RBF-SVM Classification on Microarray Datasets and the P Value of Wilcoxon Sign Rank Test (2) Table S2—The Rank of Macro-AUC and Micro-AUC of Rprop+ NN Classification on Microarray Datasets and the P Value of Wilcoxon Sign Rank Test (3) Table S3—The parameter selection and tunning range (4) Figure S1—The regression fitting results of the classification accuracy of gene subsets with different DB values (5) Figure S2—Visualization results of each dataset. [file 12859_2023_5267_MOESM1_ESM.docx]

**Computational Complexity Analysis**

Since the proposed model is an embedded method, we discuss the computational complexity separately for each algorithm in it.

First, we estimate the intrinsic dimensionality of the dataset using MLE, which requires searching the k-nearest neighbors of each data points. According to the ider [1], an R package for implementing MLE, the KD Tree algorithm is used for k-nearest neighbors search with a time complexity of $O(\log n)$ [2]. The result of the MLE gives the target space dimension $d$ of Isomap. Another parameter of Isomap Another parameter $k$ of Isomap is determined by the grid search. The DB-Index is computed for each candidate $k$ value with a time complexity of $O(p)$ [3], where $p$ is the dimension of input dataset.

The parameters of GA are given, e.g., individual size, population size, so the complexity of GA depends only on the calculation of fitness function. According to the definition of Iso-GA, the complexity of the fitness function computation is the sum of complexity of Isomap and DB-Index. The time complexity of Isomap is $O\left( n^{3} \right)$, as mentioned in the supplemental information of [4], and the complexity of DB-Index is $O(d)$. Here, because $d$ is given by MLDE, the complexity is reduced to $O(1)$ which can be ignored.

The two classifiers used in this work are not part of our proposed Iso-GA method, and can be replaced by other classifiers, so they are not being discussed here.

Therefore, the computational complexity of the proposed Iso-GA is $O\left( n^{3} \right)$, and the complexity of parameter selection for Isomap is $O\left( \log n \right)+O(p)$.

**Reference**

1. Hino H. ider: Intrinsic Dimension Estimation with R. R Journal. 2017 Dec;9(2):329. https://cran.r-project.org/package=ider

2. Beygelzimer A, Kakadet S, Langford J, Arya S, Mount D, Li S. FNN: fast nearest neighbor search algorithms and applications. R package version. 2022;1(1):1–17. https://cran.r-project.org/package=FNN

3. Muravyov S, Antipov D, Buzdalova A, Filchenkov A. Efficient Computation of Fitness Function for Evolutionary Clustering. MENDEL. 2019 Jun 24;25(1):87–94. https://mendel-journal.org/index.php/mendel/article/view/83

4. Bartenhagen C, Klein HU, Ruckert C, Jiang X, Dugas M. Comparative study of unsupervised dimension reduction techniques for the visualization of microarray gene expression data. Vol. 11, BMC Bioinformatics. 2010.

| **Table S1** The Rank of Macro-AUC and Micro-AUC of RBF-SVM Classification on Microarray Datasets and the P Value of Wilcoxon Sign Rank Test   \| Dataset \|  \| Iso-GA \| MDS-GA \| GA \| \| --- \| --- \| --- \| --- \| --- \| \| Breast \| Ma-rank \| 1 \| 2 \| 3 \| \|  \| p \| - \| 0.22 \| 0.22 \| \|  \| Mi-rank \| 1 \| 2 \| 3 \| \|  \| p \| - \| 0.22 \| 0.22 \| \| CNS \| Ma-rank \| 3 \| 1 \| 2 \| \|  \| p \| - \| 0.15 \| 0.13 \| \|  \| Mi-rank \| 3 \| 1 \| 2 \| \|  \| p \| - \| 0.10 \| 0.16 \| \| Colon \| Ma-rank \| 2 \| 1 \| 3 \| \|  \| p \| - \| 0.61 \| 0.18 \| \|  \| Mi-rank \| 2 \| 1 \| 3 \| \|  \| p \| - \| 0.5 \| 0.09 \| \| Leukemia \| Ma-rank \| 1 \| 2 \| 3 \| \|  \| p \| - \| 0.5 \| 0.5 \| \|  \| Mi-rank \| 1 \| 2 \| 3 \| \|  \| p \| - \| 0.19 \| 0.5 \| \| Lung \| Ma-rank \| 2 \| 1 \| 3 \| \|  \| p \| - \| 0.22 \| 0.22 \| \|  \| Mi-rank \| 2 \| 1 \| 3 \| \|  \| p \| - \| 0.16 \| 0.16 \| \| Lymphoma \| Ma-rank \| 1.5 \| 1.5 \| 3 \| \|  \| p \| - \| - \| 0.5 \| \|  \| Mi-rank \| 1 \| 2 \| 3 \| \|  \| p \| - \| 0.5 \| 0.5 \| \| MLL \| Ma-rank \| 3 \| 2 \| 1 \| \|  \| p \| - \| 0.5 \| 0.5 \| \|  \| Mi-rank \| 2 \| 3 \| 1 \| \|  \| p \| - \| 0.5 \| 0.4 \| \| SRBCT \| Ma-rank \| 1/3 \| 1/3 \| 1/3 \| \|  \| p \| - \| - \| - \| \|  \| Mi-rank \| 2 \| 1 \| 3 \| \|  \| p \| - \| 0.9 \| 0.5 \| \| Rank Sum \|  \| 27.8 \| 23.8 \| 39.3 \|   Ma-rank: Ranking of Macro-AUC  Mi-rank: Ranking of Micro-AUC |
| --- | --- | --- | --- | --- | --- | --- | --- | --- | --- | --- | --- | --- | --- | --- | --- | --- | --- | --- | --- | --- | --- | --- | --- | --- | --- | --- | --- | --- | --- | --- | --- | --- | --- | --- | --- | --- | --- | --- | --- | --- | --- | --- | --- | --- | --- | --- | --- | --- | --- | --- | --- | --- | --- | --- | --- | --- | --- | --- | --- | --- | --- | --- | --- | --- | --- | --- | --- | --- | --- | --- | --- | --- | --- | --- | --- | --- | --- | --- | --- | --- | --- | --- | --- | --- | --- | --- | --- | --- | --- | --- | --- | --- | --- | --- | --- | --- | --- | --- | --- | --- | --- | --- | --- | --- | --- | --- | --- | --- | --- | --- | --- | --- | --- | --- | --- | --- | --- | --- | --- | --- | --- | --- | --- | --- | --- | --- | --- | --- | --- | --- | --- | --- | --- | --- | --- | --- | --- | --- | --- | --- | --- | --- | --- | --- | --- | --- | --- | --- | --- | --- | --- | --- | --- | --- | --- | --- | --- | --- | --- | --- | --- | --- | --- | --- | --- | --- | --- | --- | --- | --- |

| **Table S2** The Rank of Macro-AUC and Micro-AUC of Rprop+ NN Classification on Microarray Datasets and the P Value of Wilcoxon Sign Rank Test   \| Dataset \|  \| Iso-GA \| MDS-GA \| GA \| \| --- \| --- \| --- \| --- \| --- \| \| Breast \| Ma-rank \| 2 \| 3 \| 1 \| \| p \| - \| 0.5 \| 0.3 \| \| Mi-rank \| 1 \| 2 \| 3 \| \| p \| - \| 0.5 \| 0.4 \| \| CNS \| Ma-rank \| 3 \| 1 \| 2 \| \| p \| - \| 0.06 \| 0.8 \| \| Mi-rank \| 3 \| 1 \| 2 \| \| p \| - \| 0.2 \| 0.4 \| \| Colon \| Ma-rank \| 1 \| 3 \| 2 \| \| p \| - \| 0.3 \| 0.3 \| \| Mi-rank \| 1 \| 3 \| 2 \| \| p \| - \| 0.2 \| 0.2 \| \| Leukemia \| Ma-rank \| 1 \| 2 \| 3 \| \| p \| - \| 0.2 \| 0.5 \| \| Mi-rank \| 1 \| 2 \| 3 \| \| p \| - \| 0.4 \| 0.2 \| \| Lung \| Ma-rank \| 1 \| 2 \| 3 \| \| p \| - \| 0.3 \| 0.4 \| \| Mi-rank \| 2 \| 1 \| 3 \| \| p \| - \| 0.6 \| 0.4 \| \| Lymphoma \| Ma-rank \| 3 \| 1.5 \| 1.5 \| \| p \| - \| 0.5 \| 0.5 \| \| Mi-rank \| 3 \| 1.5 \| 1.5 \| \| p \| - \| 0.3 \| 0.3 \| \| MLL \| Ma-rank \| 1 \| 3 \| 2 \| \| p \| - \| 0.5 \| 0.8 \| \| Mi-rank \| 1 \| 3 \| 2 \| \| p \| - \| 0.6 \| 0.15 \| \| SRBCT \| Ma-rank \| 2 \| 1 \| 3 \| \| p \| - \| 0.9 \| 0.09 \| \| Mi-rank \| 3 \| 1 \| 2 \| \| p \| - \| 0.5 \| 0.15 \| \| Ranking Sum \|  \| 29 \| 31 \| 36 \| |
| --- | --- | --- | --- | --- | --- | --- | --- | --- | --- | --- | --- | --- | --- | --- | --- | --- | --- | --- | --- | --- | --- | --- | --- | --- | --- | --- | --- | --- | --- | --- | --- | --- | --- | --- | --- | --- | --- | --- | --- | --- | --- | --- | --- | --- | --- | --- | --- | --- | --- | --- | --- | --- | --- | --- | --- | --- | --- | --- | --- | --- | --- | --- | --- | --- | --- | --- | --- | --- | --- | --- | --- | --- | --- | --- | --- | --- | --- | --- | --- | --- | --- | --- | --- | --- | --- | --- | --- | --- | --- | --- | --- | --- | --- | --- | --- | --- | --- | --- | --- | --- | --- | --- | --- | --- | --- | --- | --- | --- | --- | --- | --- | --- | --- | --- | --- | --- | --- | --- | --- | --- | --- | --- | --- | --- | --- | --- | --- | --- | --- | --- | --- | --- | --- | --- | --- | --- | --- | --- | --- | --- | --- | --- | --- | --- | --- | --- |

**Table S3**
The parameter selection and tunning range

|  | **Parameters of algorithms** | **Cross-validation** | **Parameters of classifiers** |
| --- | --- | --- | --- |
| **Iso-GA** | **Isomap**:  k tuned in [5,20]  **GA**:  Individual size: 30  The number of generations: 100  The number of elitisms: 5  (Other parameters are default of kofnGA package)  **MLE** (for calculating d of Isomap):  k1: 20  k2: 30 | **Nested cross-validation**:  outer side:  5-fold  inner side:  bootstrap 10 times | **RBF-SVM**:  Sigma (kerel width) tuned in [0.001, 0.011, 0.021, …, 0.091)  C (cost of constraints violation) tuned in [1, 2, 3, …, 10]  **Rprop+ NN**:  hn1 tuned in [10, 12, 14, …, 30]  hn2 tuned in [4, 6, 8, …, 20] |
| **MDS-GA** | Same with GA in Iso-GA | Same with Iso-GA | Same with Iso-GA |
| **GA** | Same with GA in Iso-GA | Same with Iso-GA | Same with Iso-GA |
| **MBEGA** | * | * | * |
| **CER-ABC** | **ABC**:  population size: 40  maximum number of iterations: 2000  limit: 100  $th$ tuned in {0.1, 0.3, 0.5, 0.7, 0.9} | 5-fold cross-validation | Same with Iso-GA |

*: The result of this method is directly used the published results without implementing this method, so no parameter setting is required.

|   **Figure S1** The regression fitting results of the classification accuracy of gene subsets with different DB values. In the order from top to bottom and left to right: Breast, CNS, Colon, Leukemia, Lung, Lymphoma, MLL, SRBCT. |
| --- |

| 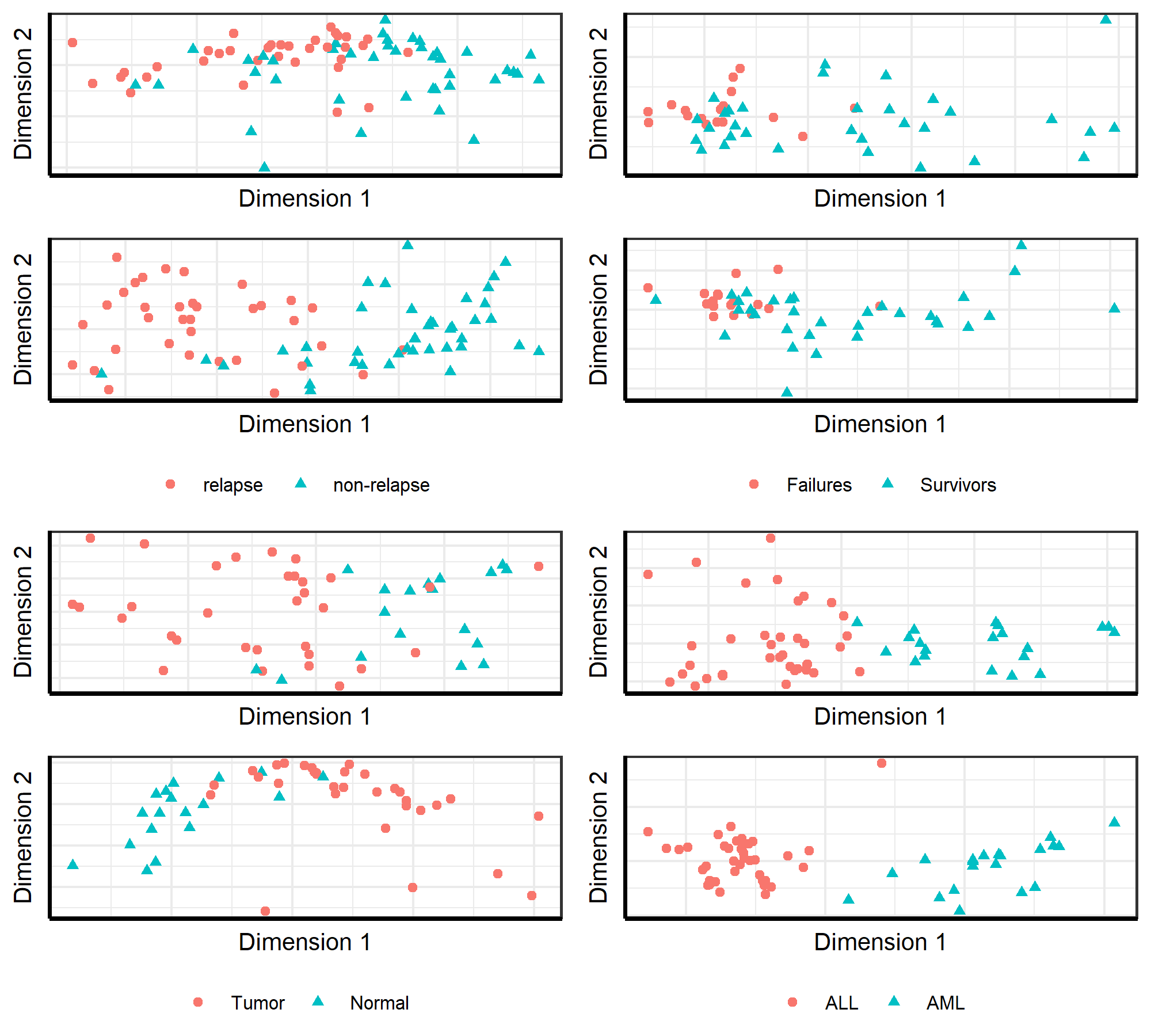  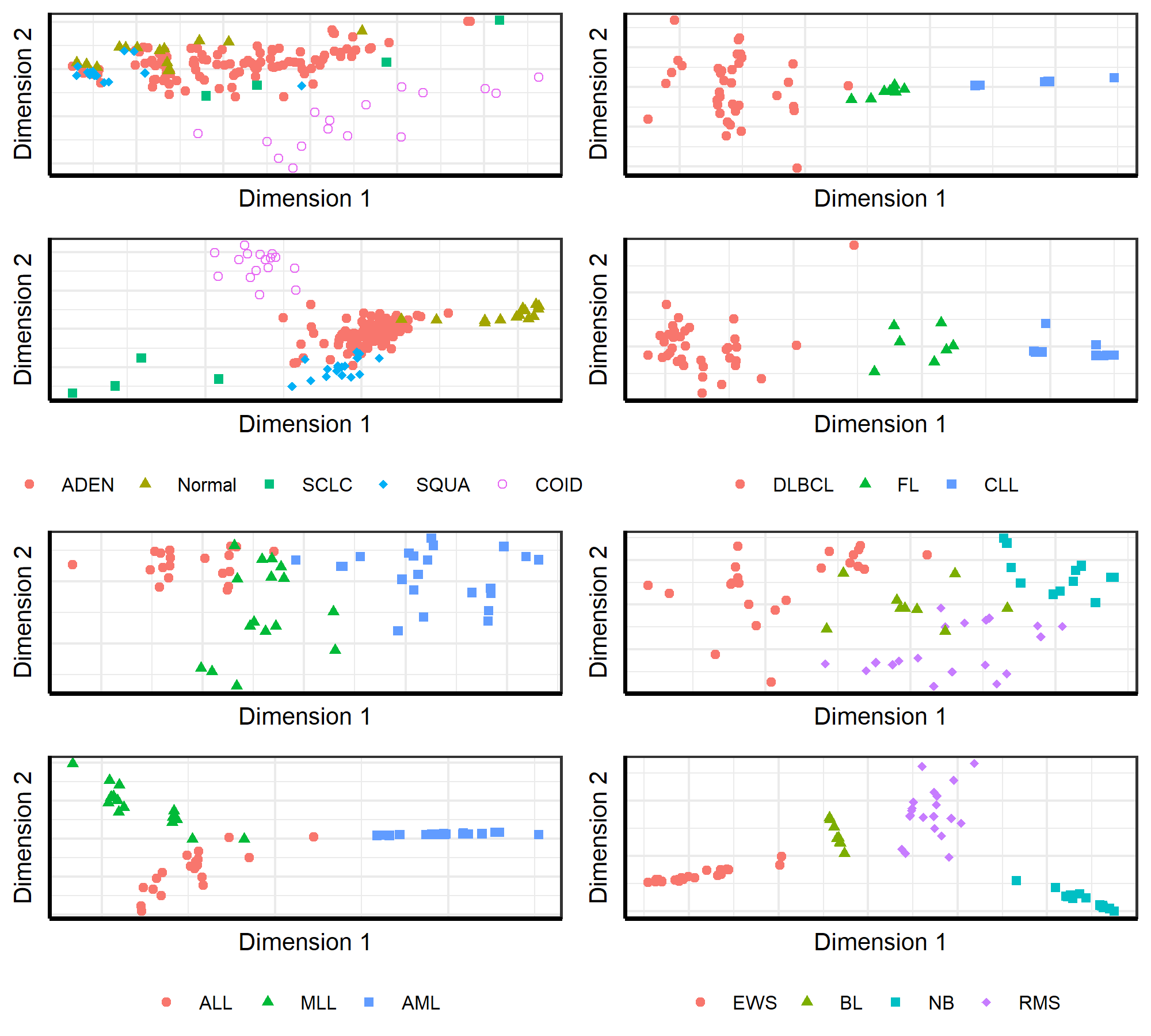  **Figure S2** Visualization results of each dataset. In the order from top to bottom and left to right: Breast, CNS, Colon, Leukemia, Lung, Lymphoma, MLL, SRBCT. |
| --- |
